# Impaired everyday memory associated with encephalopathy of severe malaria: the role of seizures and hippocampal damage

**DOI:** 10.1186/1475-2875-8-273

**Published:** 2009-12-01

**Authors:** Michael Kihara, Julie A Carter, Penny A Holding, Faraneh Vargha-Khadem, Rod C Scott, Richard Idro, Greg W Fegan, Michelle de Haan, Brian GR Neville, Charles RJC Newton

**Affiliations:** 1 The Centre for Geographical Medicine Research (Coast), Kenya Medical Research Institute, Kilifi, Kenya; 2 Centre for International Health and Development, University College London Institute of Child Health, UK; 3 Case Western Reserve University, International Centre for Behavioural Development, USA; 4 Developmental Cognitive Neuroscience Unit, University College London Institute of Child Health, UK; 5 Neurosciences Unit, Institute of Child Health, University College London, London, UK; 6 Department of Paediatrics and Child Health, Mulago Hospital/Makerere University, Kampala, Uganda; 7 Infectious Disease Epidemiology Unit, Department of Epidemiology and Population Health, London School of Hygiene & Tropical Medicine, UK; 8 Clinical Research Unit, Infectious Disease Epidemiology Unit, Department of Epidemiology and Population Health, London School of Hygiene & Tropical Medicine, UK

## Abstract

**Background:**

Seizures are common in children admitted with severe falciparum malaria and are associated with neuro-cognitive impairments. Prolonged febrile seizures are associated with hippocampal damage and impaired memory. It was hypothesized that severe malaria causes impaired everyday memory which may be associated with hippocampal damage.

**Methods:**

An everyday memory battery was administered on 152 children with cerebral malaria (CM) (mean age, 7 y 4 months [SD 13 months]; 77 males) 156 children (mean age, 7 y 4 months [SD, 14 months]; 72 males) with malaria plus complex seizures (MS) and 179 children (mean age, 7 y 6 months [SD, 13 months]; 93 males) unexposed to either condition.

**Results:**

CM was associated with poorer everyday memory [95% CI, -2.46 to -0.36, p = 0.004] but not MS [95% CI, -0.91 to 1.16, p = 1.00] compared to unexposed children. Children with exposure to CM performed more poorly in recall [95% CI, -0.79 to -0.04, p = 0.024] and recognition subtests [95% CI, -0.90 to -0.17, p = 0.001] but not in prospective memory tests compared to controls. The health factors that predicted impaired everyday memory outcome in children with exposure to CM was profound coma [95% CI, 0.02 to 0.88, p = 0.037] and multiple episodes of hypoglycaemia [95% CI, 0.05 to 0.78, p = 0.020], but not seizures.

**Discussion:**

The findings show that exposure to CM was associated with a specific impairment of everyday memory. Seizures commonly observed in severe malaria may not have a causal relationship with poor outcome, but rather be associated with profound coma and repeated metabolic insults (multi-hypoglycaemia) that are strongly associated with impaired everyday memory.

## Background

*Plasmodium falciparum *is the most common parasitic infection of the central nervous system. Every year, over two billion people are infected and over a million die [1]. Children living in sub-Saharan Africa bear the brunt of the disease in which the most common neurological complications are seizures and impaired consciousness i.e. cerebral malaria (CM) [2]. These neurological manifestations are associated with neurocognitive sequelae [3]. Previous reports show that between 5 and 26% of children exposed to severe falciparum malaria have cognitive impairment, in particular affecting language, executive functioning [4-6] and memory [3]. It is important to establish whether cognitive and memory deficits in children with cerebral malaria are a consequence of seizures, the underlying CM or a combination of factors as this would guide therapeutic strategies that aim to minimize adverse memory outcomes following CM.

Seizures are a common feature in children with severe or cerebral falciparum malaria; on admission, over 80% have a history of seizures and these recur in over 60% during the course of admission [7]. In the context of acute malaria, it is unclear what proportion of seizures are caused by fever, by metabolic stress or by the sequestration of the parasites within the vasculature of the brain [8]. Although fever commonly occurs in malaria, the seizures observed in children admitted to hospital are not regarded as simple febrile seizures, since over 50% occur when the child is afebrile [9], and they are often complex (i.e. focal, prolonged or repetitive) [10]. Prolonged febrile seizures not precipitated by malaria, have been associated with hippocampal injury [11-13], which may present later as temporal lobe seizures associated with mesial temporal sclerosis (MTL), impaired memory [14], and/or behavioural problems [15]. It is therefore possible that prolonged seizures within the context of acute malaria could also result in hippocampal injury associated with later episodic memory impairments (i.e. memory for everyday episodes) [16,17]. Direct evidence linking cerebral malaria to hippocampal damage is confined to two single cases, one with pathology following surgery [18] and the other a cognitive study [19]. It remains uncertain in these cases whether the MTS was present prior to the episode of malaria, or whether it was acquired during the acute episode either as a function of the seizures or of direct effects of the parasite. There is, however, little data available that examines the association of severe malaria and hippocampal function, particularly in African children who bear the brunt of the disease.

The hypothesis was that children exposed to severe malaria may have hippocampal injury and therefore will have impaired everyday memory. The aims of the current study therefore were to;

1. Define memory impairments associated with cerebral malaria in African children

2. Investigate whether memory outcomes are worse in children with seizures

3. To evaluate clinical risk factors for memory impairments in children exposed to malaria

## Methods

The study took place in the catchment area of Kilifi District Hospital (KDH), a malaria-endemic area on the coast of Kenya, where the population is largely dependent upon subsistence farming. Three groups of children were examined: those discharged following recovery from cerebral malaria (CM) (defined as a Blantyre coma score (BCS) of less or equal to 2 for 4 or more hours, a peripheral falciparum malaria parasitaemia and exclusion of other causes of encephalopathy [20]), Children discharged following malaria plus complex seizures (MS) (defined as more than two seizures within 24 hours or focal or prolonged for more than 30 minutes but did not lapse into coma), and a comparison group of children unexposed to either condition living in the same community.

The children had a neurological examination and underwent cognitive testing as part of a larger study, the results of which have been reported elsewhere [4,21,22]. Children were excluded if their parents or guardians refused informed written consent or the child refused verbal informed consent. The Kenya Medical Research Institute Scientific Steering and Ethical Review Committees approved the study.

### Memory assessment

The Kilifi Creek Behavioural Memory Test for Children (KCBMT), an everyday memory battery adapted from the Rivermead Behavioural Memory Test (RBMT-C) [23] was administered. It was developed to examine everyday memory i.e. memory needed to lead everyday life independently. It is composed of sub-tests of recall (memory of past events), recognition (memory of past events given clues) and prospective memory (remembering to carry out some action in the future). The RBMT-C was modified to make the test materials more familiar to the local children. Objects familiar to the local children and photographs of local people were used to replace those in the RBMT-C. The content of the orienting questions were also changed to reflect local experience.

In order to ensure equal weighting, performance on each subtest was ranked on scale ranging from 0 (impaired), 1 (borderline) to 2 (normal), yielding a total "profile" score. In addition, three composite scores were created by summing the rankings of each of the following measures: (i) delayed recall subtests (i.e. story, route, message recall) (ii) recognition subtests (i.e. picture recognition and face recognition, referred to as 'recognition memory') and (iii) prospective memory (appointment, name, hidden object). Impaired everyday memory was defined as 2 standard deviations below the age-group total profile score.

### Underlying Cognitive Measures

In the present study, it was not possible to use a standardized intelligence (IQ) tests since none are available appropriate for use in the target population. Instead, the cognitive language domains of a speech and language test to control for underlying cognitive abilities [24] were used. In particular, receptive vocabulary (Picture Vocabulary Test) and expressive language are used as a proxy for IQ.

### Other Background Factors (Health and Environmental)

These factors were age (6, 7, 8 or 9 years) sex (male or female), socio-economic status (high or low), schooling (yes or no) and IQ (high or low). Clinical factors were denoted as either 'yes' or 'no' and included; profound coma (BCS = 0 or 1), prolonged coma (> 24 hours), deep breathing, hypoglycaemia (blood glucose < 2.2 mmol/L), multiple episodes of hypoglycaemia (≥ 3 episodes), severe anaemia (Hb < 5 g/dl), history of seizures before admission, prolonged seizures (> 30 minutes), multiple seizures (> 3 seizures), height for age (stunting) (z-score < -3SD) and weight for age (wasting) (z-score < -3SD). These were all obtained from the child's hospital records at admission.

### Statistical analysis

Analysis was performed using SPSS for Windows version 15 (Chicago, Illinois, USA). The total profile score of the KBMTC was computed for each child and it represented their everyday memory. The everyday memory outcome and three composite measures of recall, recognition and prospective memory, of the three diagnostic groups i.e. CM, MS and unexposed children, were compared using analysis of covariance (ANCOVA) with age and schooling as covariates. The Bonferroni adjustment for multiple comparisons was used in the post hoc comparisons (t-tests) for simple effects. Statistical significance was set at p < 0.05. A multivariable logistic regression model, with impaired memory as a dependent variable, was used to determine those factors that are independently associated with poor everyday memory performance.

## Results

All the children were born between 1991 and 1995 and were between 6:0 and 9:11 years during assessment which took place a minimum of 20 months post-discharge. A total of 152 children with a history of CM (mean age 7 y 4 months [SD 13 months]; 77 males) and 156 with a history of MS (mean age, 7 y 4 months [SD, 14 months]; 72 males) were selected from the admission database at the KDH. A random sample of 179 children unexposed to either condition (mean age, 7 y 6 months [SD, 13 months]; 93 males) was sampled from the census database of children living in the same study area.

### Underlying Cognitive Abilities (Cognitive language measures)

An analysis of variance of the cognitive language measures by diagnosis revealed significant differences between groups for both receptive vocabulary (PVT) [F (2, 483) = 5.980, p = 0.003] and expressive language [F (2, 483) = 6.689, p = 0.001]. The mean receptive vocabulary score in CM was 20.6 (95% CI, 20.1 to 21.2), in MS was 21.5 (95% CI, 21.0 to 22.0) and in controls was 22.0 (95% CI, 21.5 to 22.5). After Bonferroni adjustment, there was a significant difference between CM and controls (p = 0.002). There was no significant difference between MS and controls or between the two malaria groups. The mean expressive language score in CM was 34.9 (95% CI, 34.1 to 35.8), in MS was 36.7 (95% CI, 35.8 to 37.5) and in controls was 37.0 (95% CI, 36.2 to 37.7). The Bonferroni adjustment for multiple comparisons there was applied and there was significant difference between CM and controls (p = 0.002) and between CM and MS (p = 0.013).

### Everyday Memory (Total Profile Score)

The diagnostic groups differed significantly by schooling [F (2, 484) = 4.904, p = 0.008], marginally by age (p = 0.069) but however did not differ by sex (p = 0.702) or socioeconomic status (p = 0.957).

**Figure 1:**
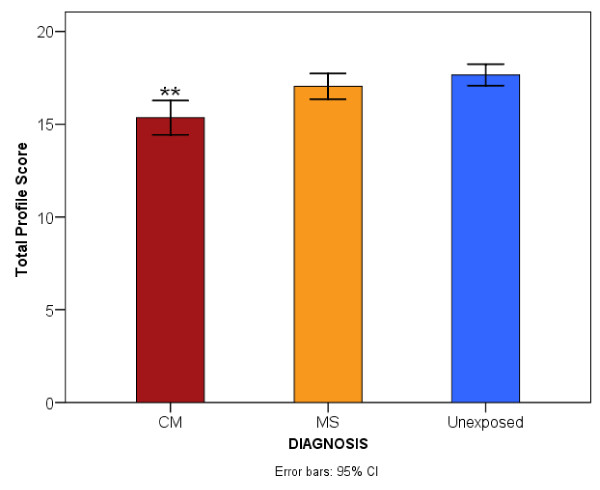
**Comparison of the mean total profile score of the children by diagnosis**. "CM represents cerebral malaria, MS is malaria plus seizures. Significant level **P < 0.01".

The analysis of covariance (ANCOVA) with total profile score (everyday memory) as dependent variable, diagnostic group (i.e CM, MS and unexposed) as fixed factor and, age, cognitive language scores and schooling as covariates revealed that diagnostic category was associated with impaired everyday memory [F (2, 482) = 7.377, p = 0.001]. Children with a history of CM had a mean total profile score of 15.7 (95% CI, 15.1 to 16.4), those with MS had 17.1 (95% CI, 16.5 to 17.8) and unexposed children 17.2 (95% CI, 16.6 to 17.8). *Post-hoc *comparisons for simple effects of disease exposure revealed significantly poorer performance in those children with a history of CM (p = 0.004) but not of those exposed to MS (p = 1.000) compared to unexposed children. Children with a history of CM performed significantly poorer than those exposed to MS (p = 0.002). Figure 1 illustrates the mean total profile scores for the three diagnostic groups.

### Children's performance on composite measures

**Figure 2:**
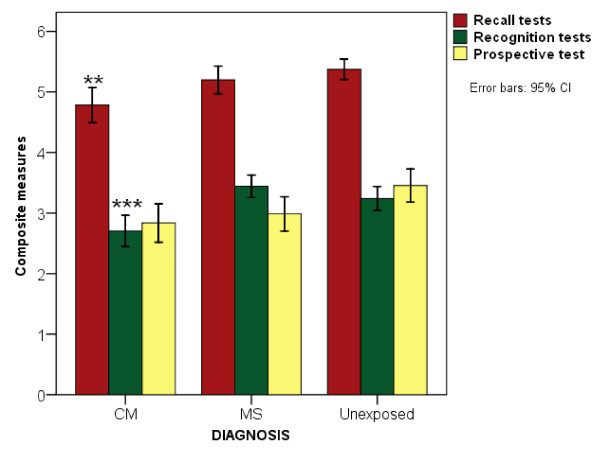
**Comparison of the mean composite scores of the children by diagnosis**. "Significance level: ** p < 0.01, *** p < 0.001. Recall memory composed of delayed story recall, delayed route recall and memory recall; Recognition memory composed of picture recognition and face recognition; Prospective items composed of remember appointment, remember name and remember hidden object."

An ANCOVA of the composite measures revealed statistically significant group differences in recall memory [F (2, 482) = 4.128, p = 0.017] and recognition memory [F (2, 482) = 11.687, p < 0.001] but not in prospective memory items [p = 0.552, *ns*] (Figure 2). The mean scores for recall, recognition and prospective memory for the children with a history of CM [4.9 (95% CI, 4.7 to 5.1), 2.8 (95% CI, 2.6 to 3.0) and 2.9 (95% CI, 2.6 to 3.2) respectively], MS [5.2 (95% CI, 5.0 to 5.4), 3.4 (95% CI, 3.2 to 3.6) and 3.0 (95% CI, 2.7 to 3.2)] and unexposed children [5.3 (95% CI, 5.0 to 5.5), 3.2 (95% CI, 2.9 to 3.3) and 3.3 (95% CI, 3.0 to 3.6)] were compared.

*Post-hoc *pair-wise comparisons of diagnostic groups again revealed that these differences were largely accounted for by poorer performance in children with a history of CM compared to unexposed children. In both recall and recognition memory children with exposure to CM performed significantly more poorly (p = 0.024 and p = 0.001 respectively). The difference between children with a diagnosis of CM and those with a history of MS only reached significance for recognition memory (p < 0.001) but not recall (p = 0.064, *ns*). The difference between children exposed to MS and unexposed children did not reach significance on either composite measure [recall p = 1.00, *ns *and recognition p = 0.552, *ns*].

### Background factors independently associated with performance in everyday memory

Univariable analysis showed that diagnostic group, nutrition, schooling, cognitive language score (used as IQ proxy), duration of seizures and number of seizures (Table 1) were associated with impaired everyday memory. Children with CM performed four times more poorly than unexposed children [OR 4.5, 95% CI 1.692 to 12.913]. Having a seizure that lasted at least five minutes increased the odds for poor performance approximately three-fold [OR 2.8, 95% CI 1.049 to 7.626], as did those who had seizures that lasted longer than 30 minutes [OR 2.8, 95% CI 1.064 to 7.740]. Children who had more than 9 seizures performed significantly poorer than those without seizures [OR 4.0, 95% CI 1.426 to 11.291]. These factors were then entered into a multivariable logistic regression analysis to determine which factors independently predicted poor outcome. In the full model, a significant association remained between diagnostic category (i.e. CM) and impaired everyday memory [B = -1.17, Wald χ^2 ^= 4.650, 95% CI 0.108 to 0.899, p = 0.030]. Additional factors with a significant association were nutrition [B = 1.35, Wald χ^2 ^= 9.373, 95% CI 1.628 to 9.215, p = 0.006] and schooling [B = -1.55, Wald χ^2 ^= 10.612, 95% CI 0.083 to 0.539, p = 0.001]. The number of seizures or seizure duration did not independently predict everyday memory.

**Table 1:** Univariate analysis of health factors and seizure variables on impaired everyday memory of children

Variable	P-value	OR associated with impairment	95% CI
Sex (female)	0.125	1.803	0.850 to 3.826
Age			
7 years	0.652	0.791	0.285 to 2.193
8 years	0.836	0.897	0.323 to 2.494
9 years	0.902	1.062	0.407 to 2.771
Diagnosis			
Cerebral malaria	**0.003**	4.675	1.692 to 12.913
Malaria plus seizures	0.184	2.131	0.699 to 6.498
Nutrition			
weight for age	** < 0.001**	5.434	2.373 to 12.445
Height for weight	** < 0.001**	4.861	2.249 to 10.507
SES (high)	0.235	0.618	0.280 to 1.367
Schooling	** < 0.001**	0.154	0.062 to 0.381
Cognitive language (IQ proxy)*	** < 0.001**	26.444	0.781 to 0.919
Epilepsy	0.438	0.549	0.121 to 2.501
Seizure duration (at least 5 min)	**0.040**	2.829	1.049 to 7.626
Seizure duration (5> × >30 min)	0.936	0.957	0.324 to 2.821
Seizure duration (more than 30 min)	**0.037**	2.870	1.064 to 7.740
Multiple seizures (less than 3)	0.557	1.373	0.477 to 3.948
Multiple seizures (between 4 and 9)	0.246	2.063	0.607 to 7.012
Multiple seizures (more than 9)	**0.008**	4.012	1.426 to 11.291

*Cognitive language scores were used as a proxy for IQ. Age was compared to children aged 6 years old and diagnosis compared to unexposed children

### Clinical health factors of CM children and everyday memory

Backward stepwise multivariable logistic regression analysis with impaired everyday memory as a dependent variable to examine the health factors associated with poor outcome in the children with a diagnosis of CM was used. The clinical factors were prolonged seizures, multiple seizures, deep coma, profound coma, severe hypoglycaemia, multiple hypoglycaemia, stunting (height for age) and wasting (weight for age). The results showed that impaired everyday memory was associated with profound coma [(B = -2.58, Wald χ^2 ^= 7.210, 95% CI 0.02 to 0.88, p = 0.007] and multi-hypoglycaemic episodes [B = -1.62, Wald χ^2 ^= 5.397, 95% CI 0.05 to 0.78, p = 0.020]. Again, the seizures variables did not significantly affect the outcome of children who had a history of CM.

## Discussion

This study investigated the association between severe falciparum malaria in children and everyday memory impairment. To understand better the pathogenesis of cognitive impairment in severe malaria, we were interested in exploring whether impairment in everyday memory is hippocampal dependent. The results showed that everyday memory was significantly impaired in children with a history of CM. Children exposed to CM not only performed significantly more poorly on the total profile score but also on recall and recognition sub-tests of the everyday memory battery. It may be likely that the deficit in everyday memory in children with exposure to CM was related to impaired cognition as they were significantly different with controls in the expressive and receptive language tests, which acted as markers of underlying cognitive skills. However, children with a history of MS also have poorer language scores but their performance on the everyday memory test did not differ from unexposed children.

Two factors, however, suggest that the impairment of everyday memory may not be related to an underlying involvement of the hippocampus. Firstly, a pattern of everyday memory impairment (impaired recall memory and recognition memory but preserved prospective memory) not previously associated with specific damage to the extended hippocampal system [16,25-27] is observed. Then, poor recall in children exposed to CM may suggest a retrieval deficit associated with executive dysfunction and mediated by the frontal lobes [28]. In the absence of structural neuro-imaging, which was unavailable at the study setting, it remained difficult to associate poor performance directly to hippocampal impairment.

Overall, poor everyday memory was associated with poorer nutrition, lack of schooling and a diagnosis of CM. These factors have been found to have a great impact on the cognitive development of school-age children in developing countries [22,29,30]. The factors that were associated with poor outcome in children with a history of CM in the present study, according to the logistic regression model, were profound coma and multiple episodes of hypoglycaemia. Multiple episodes of hypoglycaemia may affect everyday memory through hippocampal damage [31,32]. The hippocampus is critical to the formation of long-term memory, and may be particularly sensitive to hypoglycaemia [33]. Coma in cerebral malaria may result from either sequestration of the infected erythrocytes or from metabolic dysfunctions [34]. The pathways of memory impairments could thus be diverse depending on the pathogenesis of coma. Previous studies, both human and animal, have suggested that cerebral malaria may affect the medial temporal lobe structures of the hippocampus [18,19] causing impaired functioning.

Previous studies in Africa on the neuro-cognitive outcomes of CM have also shown that duration of coma [35,36], depth of coma [36,37], seizures [4,35-38], and hypoglycaemia [36,37] are predictors of poor outcome. Conversely, a recent prospective, but smaller study in Uganda, did not find an association between these clinical factors and poor cognitive outcome [6]. The present study suggests impairment of structures involved with recall memory, which could include hippocampal structures that may have been compromised by an interaction of seizures and coma or hypoglycaemia. However, in the absence of neuro-imaging data, it is impossible to localize which structures were impaired in children with a history of cerebral malaria. However, from the present results, it is difficult to associate seizures with poor outcome, though they may have contributed.

Cognitive outcome post-severe malaria seems to depend on a range of risk factors, some of which are dependent on the social and economic environment of the child [3]. Further, the severity of illness at admission and during hospitalization is also important. The difference in everyday memory outcome between CM and MS suggests that memory deficits have originated from sources other than seizures *per se*, possibly the mechanisms that cause prolonged and profound coma or hypoglycaemia [21]. A particularly interesting finding of the study was that multiple seizures or seizure duration did not independently predict poor outcome of everyday memory. Our findings suggest that multiple episodes of hypoglycaemia and profound coma in CM are important factors in determining impairment of everyday memory.

## Limitations of the study

The results of the present study should be interpreted within the limits of lack of data on the children's IQ. We used cognitive language scores as a proxy for IQ and assumed that nutritional and socio-economic effects were equally distributed amongst both exposed and unexposed children. There are some factors the present study was unable to ascertain. It cannot be ascertained that children with no recorded seizures post admission had seizures during their illness since they did not have continuous electroencephalographic monitoring. It is also possible that the coma was caused by severe metabolic imbalances especially multiple episodes of hypoglycaemia. Further it is possible that time spent under the glucose curve was more important than the lowest blood glucose concentration.

## Conclusion

It is concluded that CM may be associated with everyday memory deficits, but the present results don't suggest specific hippocampal involvement. The impairment is associated with profound coma and multiple hypoglycaemic episodes and these may be important determinants of poor memory post infection. Studies incorporating standardised IQ and neuroimaging would help define this consequence further. Further studies should seek to determine why the effects of the number of seizures are not linear as would be expected.

## List of abbreviations used

KCBMT: Kilifi Creek Behavioural Memory Test; CM: Cerebral malaria; MS: Malaria plus complicated seizures; RBMT-C: Rivermead Behavioural Memory Test for Children.

## Competing interests

The authors declare that they have no competing interests.

## Authors' contributions

JAC, BGRN and CRJCN were responsible for the conception of the study, its design, data collection and critical review of the manuscript. PAH was involved in the adapting of the test battery to the local context and was involved in reviewing the manuscript. RI was involved in reviewing malaria cases to fulfil the strict criteria and reviewing the draft. MdH and FVK were involved in the interpretation of the memory data and reviewing of the manuscript. RCS and GWF offered statistical help as well as reviewing the manuscript. MK wrote the initial draft of the manuscript and all other authors contributed substantially to the content. All authors have read and approved the final manuscript.

## Financial support

CRJC Newton (grant no. 070114) and Julie A. Carter (grant no. 059336) are supported by The Wellcome Trust, UK. The opinions expressed here are those of the authors and do not necessarily reflect the views of Wellcome Trust. All authors have no conflict of interest to declare.
